# Regeneration of periodontal bone defects with dental pulp stem cells grafting: Systematic Review 

**DOI:** 10.4317/jced.55574

**Published:** 2019-04-01

**Authors:** Sara Amghar-Maach, Cosme Gay-Escoda, Mª Ángeles Sánchez-Garcés

**Affiliations:** 1 Dentistry Student, Faculty of Medicine and Health Sciences, University of Barcelona, Spain; 2 MD, DDS, MS, PhD, EBOS, OMFS, Chairman and Professor of Oral and Maxillofacial Surgery, Faculty of Dentistry, University of Barcelona. Director of Master’s Degree Program in Oral Surgery and Implantology (EHFRE International University/FUCSO). Coordinator/Researcher of the IDIBELL Institute. Head of Oral and Maxillofacial Surgery Department of the Teknon Medical Center, Barcelona, Spain; 3 MD, DDS, PhD, Aggregate Professor of Oral Surgery. Master’s Degree Program in Oral Surgery and Implantology, School of Dentistry, University of Barcelona, Barcelona. Researcher of the IDIBELL Institute, Barcelona, Spain

## Abstract

**Background:**

The main objective is to evaluate the way to graft the dental pulp stem cells (DPSC) in periodontal defects that best regenerate periodontal tissues. Numerous procedures have been done to promote periodontal regeneration. Bone grafts show good gains clinically and radiographically but histologically seem to have minimal osteoinductive capacity. Another option that exceeds conventional surgery in reducing probing depth and increasing insertion is guided tissue regeneration and tissue engineering that could be an alternative approach to help in the regeneration of living functional bone and peri-dental structures.

**Material and Methods:**

A search was carried out in Cochrane, PubMed-MEDLINE and Scopus databases with keywords: “dental pulp stem cells”, “periodontal regeneration”, “guided tissue regeneration, periodontal”, “tissue regeneration”, “periodontal bone defects”, “periodontal tissue engineering” and “periodontal defect”. Inclusion criteria were articles in English, maximum 10 years old, in which DPSC were used to regenerate a periodontal defect. Exclusion criteria were studies not published in English, case reports, case series, literature reviews, and studies in which periodontal defect was caused by dental extraction.

**Results:**

Out of the 185 articles identified, 101 after excluding duplicates, of which 94 were discarded when reading the title and abstract. 7 articles were obtained for the full text reading: a case report and a case series were eliminated. The systematic review is performed with 5 animal testing studies *in vivo*. The DPSC sheets regenerate a greater amount of bone than the injection. If HGF (hepatocyte growth factor) is added, the maximum bone volume regenerated (69.3 ± 3.9 mm3; *p*<0.01) is achieved. Similar results were obtained in all carriers tested except in the controls. The periodontal ligament stem cells (PDLSC) formed more new bone, compared to DPSC (*p*<0.001). The presence of new cementum and periodontal ligament induced by CMLPs, was detected histologically but DPSC cannot achieve it alone.

**Conclusions:**

Cementum or PDL regeneration does not depend only on DPSC but on other unknown factors. PDLSC has better periodontal regeneration than DPSC. DPSC significantly favours the regeneration of periodontal bone tissue but has few advantages over other grafts. It is necessary to study which growth factors or matrices can enhance their capacity for periodontal regeneration.

** Key words:**Dental pulp, stem cells, periodontal guided tissue regeneration, periodontal bone loss.

## Introduction

Periodontitis is one of the main causes of dental loss in adults (1). Non-surgical and surgical therapies are the basis of treatment, however the way to treat patients currently is different, due to the greater pathology understanding (2).

Numerous procedures have been done to promote periodontal regeneration. Bone grafts show good gains clinically and radiographically but histologically seem to have minimal osteoinductive capacity (3,4). Another option is guided tissue regeneration (GTR) that exceeds conventional surgery in reducing probing depth and increasing insertion (5).

Tissue engineering (TE) could be considered an alternative approach and can help in the regeneration of living functional bone and peri-dental structures (4). The success of periodontal TE requires: progenitor cells capable of differentiating into osteoblasts, cementoblasts and fibroblasts, appropriate signals to modulate cell differentiation and tissue neogenesis, and a matrix to support it and facilitate these processes (6).

Dental pulp stem cells (DPSC) were the first identified human dental stem cells (7). Afterwards, stem cells were isolated from exfoliated temporal teeth, apical papilla and periodontal ligament (PDL) (6,8), that are capable to be differentiated in cementoblasts and osteoblasts (9,10). DPSCs are easily accessible in large number because can be obtained from extracted human teeth and the method of its obtention is non-invasive (11,12). It is even speculated that they can be also obtained from dental pulp with inflammation (13) or from a carious tooth (14) being able to differentiate into osteoblast-like cells forming new bone (15-17). Recent studies carried out in animal models, consider DPSCs as a potential treatment for periodontal regeneration (4,18).

The author’s main objective is to evaluate which is the carrier that allow the best results when grafting the DPSCs to regenerate periodontal bone loss (not caused by the extraction of an adjacent tooth). The secondary objective is to analyse if the DPSCs are able to restore other periodontal tissues (PDL and cementum). To reach these objectives a PICO question was maid: In periodontal bone loss defects, DPSCs grafted with a carrier have better results compared when no carrier is used in tissue engineering techniques?

## Material and Methods

A search was carried out between October and November 2016 in the Cochrane, PubMed-MEDLINE and Scopus databases. The strategy in the search of articles was made using the keywords: “dental pulp stem cells” AND “periodontal regeneration”(MeSH term) and ((“Stem Cells”[Mesh]) AND “Dental Pulp”[Mesh]) AND “Guided Tissue Regeneration, Periodontal”[Mesh]. In Scopus: “dental pulp stem cell” AND “tissue regeneration”, “periodontal bone defects” AND “dental pulp stem cell” AND “tissue regeneration”, “dental pulp stem cells” AND “periodontal regeneration”, “dental pulp stem cells” AND “tissue regeneration” AND “periodontal”, “dental pulp” AND “stem cells” AND “periodontal regeneration”, “dental pulp” AND “stem cell” AND “periodontal tissue engineering” and “periodontal defect” AND “dental pulp stem cell”. These terms were also used without MeSH in PubMed-MEDLINE.

The quality of evidence evaluation was analysed by the OSTEBA (Health Technology Assessment Service of the Basque Government Health Department) template for case series and GRADE criteria for other studies (19).

Inclusion criteria were articles published in English within the last ten years (from November 2006), in which DPSCs were used to regenerate a periodontal bone defect not caused by the previous adjacent tooth extraction, in human and animal research studies that followed ARRIVE guide criteria of the National Center for the Replacement Refinement and Reduction of Animals in Research (NC3Rs) for evaluating the quality of animal research in experimental studies *in vivo* (20). The exclusion criteria were studies not published in English, case reports, case series (less than ten) and literature reviews.

## Results

**Figure 1:**
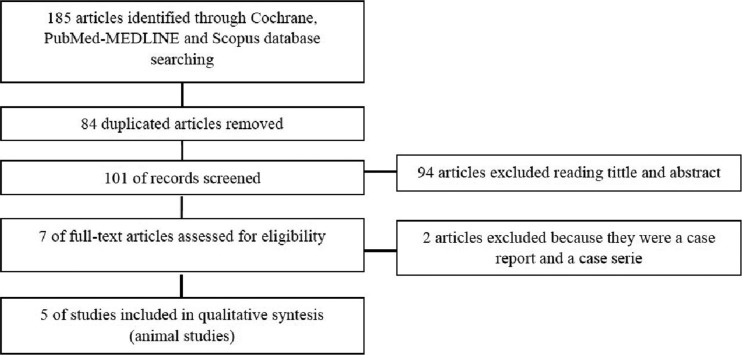
PRISMA flow diagram (21).

Out of the 185 articles initially identified, 84 duplicates were discarded, obtaining a total of 101 articles screened, of these 94 articles, 88 were excluded after reading the title and abstract because did not exactly match with the subject to be reviewed; despite coinciding with the subject, 5 reviews and an article not written in English were rejected. We obtain 7 relevant articles for full text critical reading, 2 human studies and 5 animal studies. Human studies were evaluated using the OSTEBA template for case series (18), and therefore, it was decided to discard them, since the level of evidence was low. Finally, it is decided to carry out a systematic review, as an update in preclinical research of 5 *in vivo* animal studies. Figure 1 shows PRISMA flow diagram (21). The included animal studies (22-26) follow the ARRIVE guideline criteria of the NC3Rs (20). Although one or two items were not specified, the articles were accepted for review ( Table 1).  Table 2,  Table 2 continue summarizes the main data and the comparable results of the studies included in the systematic review.

**Table 1:**
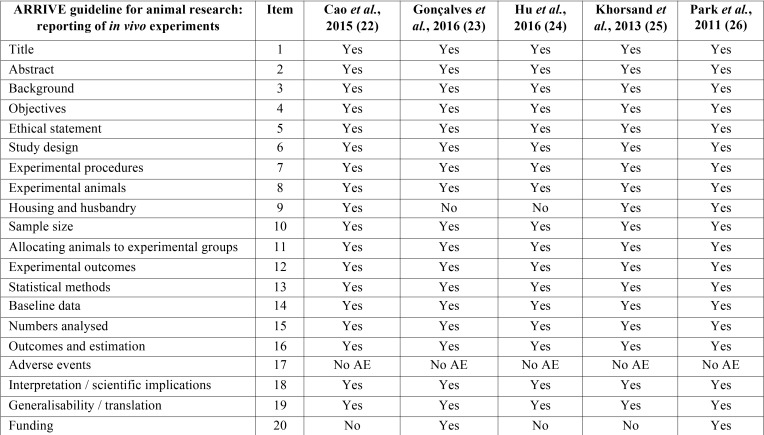
Application of the NC3Rs ARRIVE guideline. NoAE; no adverse events (20).

**Table 2:**
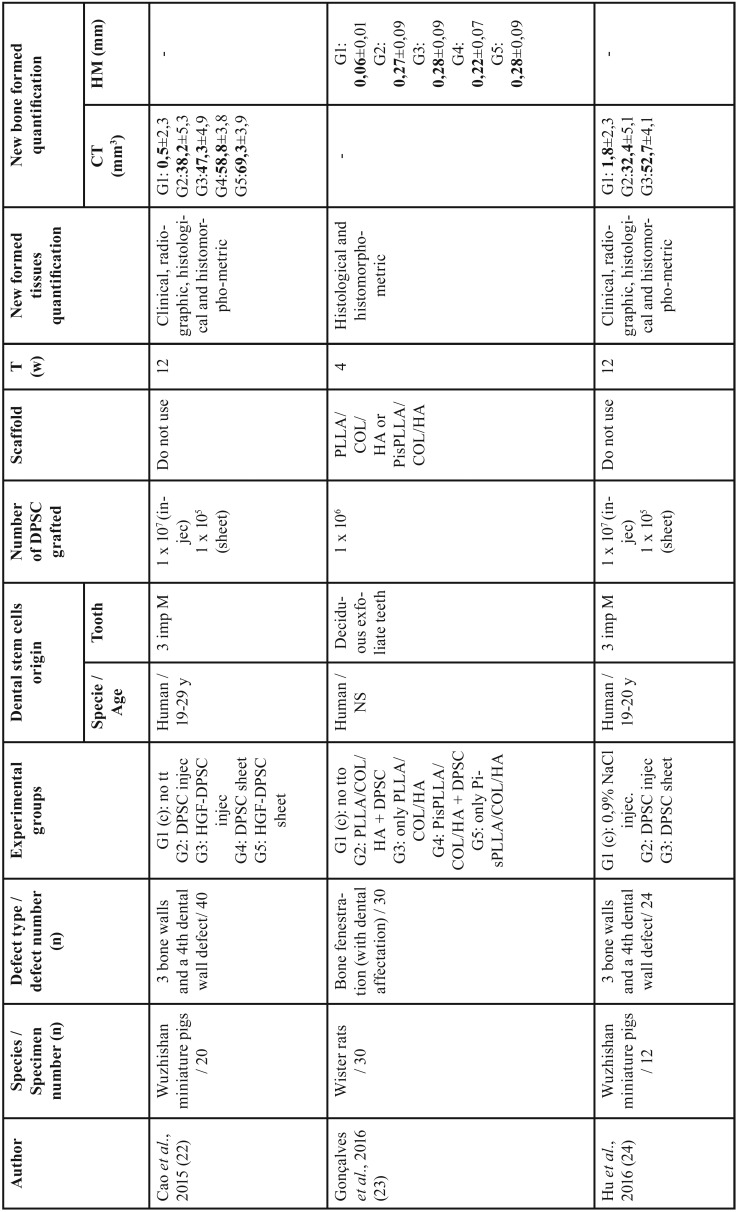
Studies included in the systematic review.

**Table 2 continue:**
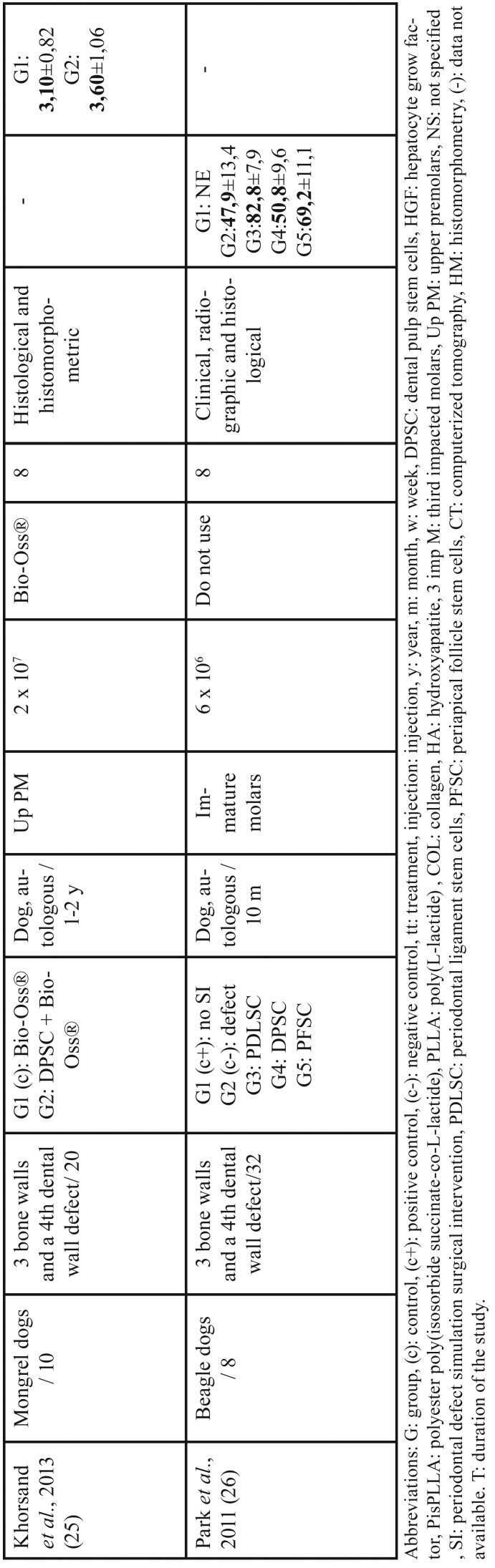
Studies included in the systematic review.

From the reviewed studies, three did not use a scaffold to graft the DPSC (22,24,26), whereas the remaining two used PLLA/COL/HA, PisPLLA/COL/HA (23) or Bio-Oss® (25) as scaffolds.

All studies take the same experimental defect of three bone walls and a fourth dental wall, except Gonçalves *et al.* (23) in which the defect is a bone fenestration with dental involvement.

In general, a significant increase in bone production was observed in the experimental groups (with DPSC), compared to the control groups in which no treatment was performed.

Cao *et al.* (22) study, used miniature pigs to create periodontal bony defects in the upper and lower first molars comparing the use of DPSC from human impacted third molars in sheets or injected, and with and without addition of hepatocyte growth factor (HGF). Grafting stem cells as sheets showed a higher amount of new bone at 12 weeks (*p* <0.01) compared to dissociated cells (DPSC injection). Hu *et al.* (24) with the same model obtained similar results. The volume of regenerated bone in the group of DPSC sheets (52.7 ± 4.1 mm3) was significantly higher than in the injection group (32.4 ± 5.1 mm3) (*p* <0.05).

In contrast, in Khorsand *et al.* (25) used 20 dogs to create a three-walled surgical defect to compare a Bio-Oss® xenograft scaffold alone or with DPSC from upper premolar. Histomorphometric analysis showed that the amount of cementum and PDL regenerated in the DPSC + Bio-Oss® group was significantly higher than the control (2.42 ± 1.40 mm and 1.77 ± 1.27 mm, respectively, *p* <0.05) (25).

In Gonçalves *et al.* (23) research, were used rats with 30 periodontal fenestration defects located in the first molar buccal root. Five experimental groups were evaluated, depending on the scaffold: poly(L-lactide), collagen and hydroxyapatite (PLLA/COL/HA) with or without DPSC, polyester poly(isosorbide succinate-co-L-lactide), collagen and hydroxyapatite (PisPLLA/COL/HA) with or without DPSC and a control group without treatment. After 4 weeks, PLLA/COL/HA or PisPLLA/COL/HA scaffolds promoted periodontal regeneration and new bone formation. An increased osteoconductive and extracellular mineralization capacity were observed for the PLLA/COL/HA scaffold, while better osteoinductive properties were associated to PisPLLA/COL/HA scaffold.

Finally, Park *et al.* (26) in periodontal defects as Cao *et al.* (22), Hu *et al.* (24) and Khorsand *et al.* (25) in a dog model, evaluated five groups: in one no intervention is performed, another to which the defect is created but not grafted and the other 3 were filled with autologous stem cell graft obtained from immature dog molars: periodontal ligament stem cells (PDLSC), DPSC and periapical follicle stem cells (PFSC). No scaffold was used to graft all the stem cells (26). PDLSC showed the best regeneration capacity of PDL, alveolar bone and cementum, as well as the innervation and irrigation of the area, which were evaluated by conventional and immunological histology, 3D micro- computed tomography (CT) and clinically (26).

## Discussion

Periodontal tissue engineering uses scaffolds associated with biomolecules and differentiable stem cells to form suitable supporting tissues. Due to the structural complexity of the PDL and the morphophysiological diversity of its tissue components, the design of scaffolds for periodontal regeneration is highly complex (23). Among the analysed studies, Bio-Oss® xenograft (25), PLLA/COL/HA or PisPLLA/COL/HA (23) are used as scaffolds. The scaffold structure and composition are extremely important in periodontal tissue engineering, since it determines the biocompatibility with the host tissues and stimulates the regeneration or inhibition of those tissues. The scaffold degradation rate should be proportional to the regenerated tissue neoformation because a rapid degradation may compromise tissue neoformation, whereas slow degradation may promote bone encapsulation or obstruction (23). In spite of this, 3 of the 5 studies analysed in this review do not used scaffold (22,24,26).

-Non-use of scaffold

•New bone formation

Cao *et al.* (22) and Hu *et al.* (24) with the same miniature pig experimental model, creates periodontal defects (three bone walls and a fourth dental wall) that were grafted with heterologous cells of human origin. Cao *et al.* (22) obtained significant bone regeneration in all the experimental groups compared to the control group (*p* <0.01). The HGF-DPSC sheet obtained the highest volume of regenerated bone (69.3 ± 3.9 mm3) and the DPSC injection the least (38.2 ± 5.3 mm3) (*p* <0.01). Therefore, it can be concluded that the addition of HGF could stimulate the bone formation (22). These results are similar to those obtained by Hu *et al.* (24). The regenerated alveolar bone volume in the group of DPSC sheets was 52.7 ± 4.1 mm3 and in the DPSC injection group 32.4 ± 5.1 mm3, data significantly higher than the volume of the control group (*p* <0.05)). In contrast, Park *et al.* (26) using the same study design in a dog model obtained almost the same results as in the negative control group (no stem cells grafted) but the bone height regenerated was significantly higher in the PDLSC group (*p* <0.0001) than in the DPSC group.

Histomorphometrically, Hu *et al.* (24) showed a percentage of new bone in the DPSC injection group was significantly greater than the control group (*p* <0.05). Therefore, the volume of new regenerated bone was higher in the DPSC sheet group than in the DPSC injection group (*p* <0.05).

If a scaffold is not used to graft, the therapeutic option that has shown the best results in terms of bone regeneration, according to the CT, is the HGF-DPSC sheet despite the stem cells were from different species.

•New periodontal ligament formation

In Cao *et al.* study (22) the new Sharpey fibers penetrates the regenerated bone tissue in all groups except for the DPSC injection and control. Similar results were observed by Hu *et al.* (24), in which there was a new union of the Sharpey fibers to the bone and the tooth in the experimental groups, unlike the control group in which it was very irregular. Park *et al.* (26) shows that the DPSC group failed to regenerate the PDL, whereas the PDLSC and the PFSC did.

•New cementum formation

Regarding cementum regeneration, based on CT, Hu *et al.* (24) observed a new layer of cementum-like tissue from the alveolar bone height to the amelo-cementum line in the experimental groups, which was not appreciated in the control group. In the study by Park *et al.* (26) as well as with regeneration of the PDL, the only groups that achieve cementum regeneration were PDLSC and PFSC.

-Bio-Oss® scaffold

•New bone formation

Xenograft scaffold used by Khorsand *et al.* (25) concluding that there is no significant difference in bone formation between the experimental and control group. However, the authors concluded that DPSC promote periodontal regeneration and claim that the DPSC-Bio-Oss® biocomplex is excellent (25).

•New periodontal ligament formation

According to a histomorphometric analysis, Khorsand *et al.* (25) obtained a mean value of new periodontal ligament of 3.30 ± 1.12 mm and 1.77 ± 1.27 mm for the experimental and control group (*p* <0.05) and concluded that the PDL is regenerated or not depending not only on the DPSC, but also on other factors which are unknown. Therefore, in terms of obturation of the defect DPSC- xenograft is physically effective, but in terms of the tissue quality obtained does not bring benefits with respect to the control.

•New cementum formation

According to histomorphometry, in the study by Khorsand *et al.* (25) the new cement formation was significantly better comparing to the control group (*p* <0.05). In addition, the regenerated cementum in the experimental group was thicker and covered a wider root surface than in the control group.

-PLLA/COL/HA or PisPLLA/COL/HA scaffold

Although PLLA and PLGA have slower degradation and better mechanical properties than collagen membranes, these materials have low cellular affinity. Therefore, the combination of synthetic and natural polymers, such as collagen, is an alternative for tissue engineering as it combines the properties of both materials. New copolymers containing isosorbide succinate and L-lactide promote increased fibroblasts adhesion and proliferation and may be a new option to explore (23).

•New bone formation

Best regeneration was seen in the groups in which a PLLA or PisPLLA/COL/HA scaffold was grafted without DPSC (*p* <0.05). In the groups in which scaffolds were grafted with DPSC the results were similar. Therefore, Gonçalves *et al.* (23) state that there is controversy in the idea that DPSC benefits cell differentiation and promotes bone and periodontal regeneration.

•New cementum formation

In the study of Gonçalves *et al.* (23), the only group in which the cementum was regenerated with complete closure was in PLLA/COL/HA without DPSC. In this study, the rat animal model and the defect type are very different from the other studies (miniature pigs or dogs), and probably this helped to have more favourable regeneration. Cao *et al.* (22) and Hu *et al.* (24) showed that the only groups in which periodontal health was recovered were those grafted into sheet form.

Park *et al.* (26) consider that autologous PDLSC and PFSC transplantation regenerate bone, cementum and PDL. PDLSC improved the insertion gain and the regeneration of periodontal tissue compared to the DPSC graft group (*p* <0.0001).

These results should be approach with caution since the origin of the stem cells was human, therefore the DPSC are of heterologous origin and the results could be influenced by an antigenicity component. In this regard, the most relevant studies to take into account for future studies with humans are the studies by Khorsand *et al.* (25) and Park *et al.* (26) because they used autologous dog DPSC, so that the results are no longer influenced by the fact that the stem cells are of a different species than the cells of the receptor.

Cao *et al.* (22) and Hu *et al.* (23) considered the regeneration with the DPSC sheet graft to be more efficient and complete than with the dissociated DPSC injection, since with the sheet it is possible to regenerate more new bone volume, as in the case of Hu *et al.* (24).

Cell sheets have been widely used and are designed to avoid the deficiencies of traditional TE as it has been shown to benefit in numerous clinical applications. A sheet of cells has a three- dimensional macrostructure that mimics the physiological functions of the extracellular matrix, thus eliminating the use of additional matrices or scaffolds. This has the advantage of avoiding the strong inflammatory responses that are induced when biodegradable matrices are degraded. In this research, the new alveolar bone and periodontal soft tissues were regenerated to almost normal levels at 12 weeks after cell sheets implantation. However, for the grafting of a cell sheet, surgery with mucoperiosteal flap elevation is required to place it on the defect (22). In contrast, for injection only a needle is inserted into the bottom of the bone defect (22,24).

There is another study in rabbits, in which they regenerate non-periodontal bone defects, combining recombinant human bone morphogenetic protein 2 (rhBMP-2) as a grow factor, DPSC and nanohydroxyapatite/collagen/poly(L-lactide) (nHAC/PLA) scaffold for the seeding, proliferation and differentiation of autologous DPSC. Therefore, they recommend this treatment as an alternative to autologous bone grafting for the clinical reconstruction of bone defects (27). This is another study that supports the better work of DPSC plus a trophic factor, since it favours cell differentiation.

As far as human studies are concerned, we find two with exactly the same analysis than this systematic review: a case report (11) and a two case report (13) but in which the bone-periodontal defect was secondary to the extraction of a third molar.

Aimetti *et al.* (11) presented a human clinical case using autologous DPSC for regeneration of a periodontal defect, evaluated with clinical, radiographic and surgical re-entry. A 56-year-old with a defect distal to a second inferior premolar, with 9 mm probing depth and 3 mm of recession, but without mobility. DPSCs they used were from a third upper molar that needed to be extracted. The dental pulp was collected with a Gracey curette and simultaneous mechanical dissociation of the dental pulp and filtration of the solution through a 50-micron filter was done. After 60 seconds of agitation, the cell suspension was collected and poured onto a collagen sponge matrix, which was placed to completely fill the infra-bone defect. This treatment got a significant clinical and radiological improvement at 6 and 12 months with filling of the intraosseous component of the defect by a bone-like tissue confirmed with surgical reentry. In this study, the DPSC are not left in culture for two or three weeks to choose the third or fourth passage as do the rest of studies, which produces enough cells (21-25). Nor do they leave a time period in which the stem cells penetrate into the pores of the scaffold (24).

A pilot study with two cases (13) in which they repaired human periodontal bone defects with autologous DPSC of inflammatory pulp tissue (from teeth with irreversible pulpitis). Although these cells have lost some of their properties, they maintain some tissue regeneration potential, being able to differentiate into osteogenic cells. Considering this pulp as a medical waste, the treatment could be more easily accepted by patients than if the pulp tissue is extracted from a healthy tooth. These stem cells were cultured for 6 days, loaded onto a β-tricalcium phosphate scaffold and grafted on the periodontal defect. After 1, 3 and 9 months, the result was clinically and radiologically evaluated. After 9 months the gingival recession, probing depth and bleeding rate slightly decreased, and furcation lesions changed from grade III to II or I (*p*<0.05). They conclude that it is a safe procedure and a possible potential treatment to be used in the future (13).

Finally, the only clinical trial with a similar theme is the study by d’Aquino *et al.* (15). Its objective was to repair an alveolar bone defect, secondary to the extraction of a lower third molar, by autologous upper molars DPSC, using a collagen sponge scaffold. In these patients, a bone and periodontal 2 or 3 walls defect is formed, with vertical loss of less than 7 mm distal to the of the distal root of the second lower molar. The study has a split-mouth design, so they used one as experimental (the collagen sponge was grafted with DPSC) and the other as a control (nothing was grafted). Three months after surgery, radiological analysis by orthopantomography confirmed that the experimental defects were completely regenerated and that the cortical level was higher than at control sites. Regarding the clinical probing depth evaluation revealed an increase in clinical insertion greater in the experimental defects than in controls 6,2 ± 2,3 mm and 4,4 ± 1,2 mm (*p*<0,05). They obtain encouraging results and demonstrate that the use of DPSC significantly favours bone repair and the collagen resorbable scaffold created an efficient and optimal biocomplex for bone regeneration. As a limitation, only 17 of the 100 patients who started the study were follow- up at the end of one year, therefore, the sample size is quite small due to the high dropout.

Although several alternatives have been proposed, ideal material is not available yet to promote periodontal regeneration in an effective, consistent and predictable way that can readily be applied clinically. The ideal material must comply simultaneously with the principles of guided tissue regeneration and TE (23).

It coincides with the review on potential stem cell-based periodontal therapy by Bassir *et al.* (8), who propose that current evidence indicates that DPSC may not be ideal multipotent stem cells for periodontal regeneration, as reported by Hynes *et al.* (4), the results are inconsistent.

According to the review of clinical use of stem cells for periodontal regeneration by Hynes *et al.* (4), the future of periodontal regeneration based on stem cells, not only with those of the dental pulp, is promising, since animal studies have shown that mesenchymal stem cells can be used for periodontal regeneration.

The time has come to move from experimental animal studies to clinical trials in humans, but first we should try to solve some important concepts such as immunogenicity, the use of autologous versus allogeneic cells, which is the source of more appropriate stem cells, the cost-effectiveness of the whole process, both for the dentist and for the patient and continue to investigate new, easy-to- obtain and low-cost carriers that facilitate their use.

## Conclusions

Keeping in mind the limitations of this work we can conclude that:

- More bone volume is produced and recovery of periodontal health is achieved when DPSC are grafted in form of cell sheets than in dissociated cell injection.

- The addition of trophic factors such as HGF, favours the cellular differentiation of DPSC, thus achieving a higher amount of regenerated bone, although in none of the studies does the stability of the bone gained over time.

- The fact that cementum and the PDL are regenerated or not depend, not only on the DPSC, but also on other unknown factors.

- There are other stem cells of dental origin, such as PDLSC that can have better results of periodontal regeneration than the DPSC.

- It is necessary to investigate which growth factors or scaffolds can better enhance the capacity of periodontal regeneration.

## References

[1] Pihlstrom BL, Michalowicz BS, Johnson NW (2005). Periodontal diseases. Lancet.

[2] Heitz-Mayfield LJ, Lang NP (2013). Surgical and nonsurgical periodontal therapy. Learned and unlearned concepts. Periodontol 2000.

[3] Bartold PM, Xiao Y, Lyngstaadas SP, Paine ML, Snead ML (2006). Principles and applications of cell delivery systems for periodontal regeneration. Periodontol 2000.

[4] Hynes K, Menicanin D, Gronthos S, Bartold PM (2012). Clinical utility of stem cells for periodontal regeneration. Periodontol 2000.

[5] Needleman IG, Worthington HV, Giedrys-Leeper E, Tucker RJ (2006). Guided tissue regeneration for periodontal infra-bony defects. Cochrane Database Syst Rev.

[6] Han J, Menicanin D, Gronthos S, Bartold PM (2014). Stem cells, tissue engineering and periodontal regeneration. Aust Dent J.

[7] Gronthos S, Mankani M, Brahim J, Robey PG, Shi S (2000). Postnatal human dental pulp stem cells (DPSCs) in vitro and in vivo. Proc Natl Acad Sci U S A.

[8] Bassir SH, Wisitrasameewong W, Raanan J, Ghaffarigarakani S, Chung J, Freire M (2016). Potential for stem cell-based periodontal therapy. J Cell Physiol.

[9] Isaka J, Ohazama A, Kobayashi M, Nagashima C, Takiguchi T, Kawasaki H (2001). Participation of periodontal ligament cells with regeneration of alveolar bone. J Periodontol.

[10] Dangaria SJ, Ito Y, Luan X, Diekwisch TG (2011). Successful periodontal ligament regeneration by periodontal progenitor preseeding on natural tooth root surfaces. Stem Cells Dev.

[11] Aimetti M, Ferrarotti F, Cricenti L, Mariani GM, Romano F (2014). Autologous dental pulp stem cells in periodontal regeneration: A case report. Int J PeriodonticsRestorativeDent.

[12] Laino G, d'Aquino R, Graziano A, Lanza V, Carinci F, Naro F (2005). A new population of human adult dental pulp stem cells: a useful source of living autologous fibrous bone tissue (LAB). J Bone Miner Res.

[13] Li Y, Zhao S, Nan X, Wei H, Shi J, Li A (2016). Stem Cell Res Ther. Repair of human periodontal bone defects by autologous grafting stem cells derived from inflammatory dental pulp tissues. Stem Cell Res Ther.

[14] Werle SB, Lindemann D, Steffens D, Demarco FF, de Araujo FB, Pranke P (2016). Carious deciduous teeth are a potential source for dental pulp stem cells. Clin Oral Investig.

[15] d'Aquino R, De Rosa A, Lanza V, Tirino V, Laino L, Graziano A (2009). Human mandible bone defect repair by the grafting of dental pulp stem/progenitor cells and collagen sponge biocomplexes. Eur Cell Mater.

[16] Karaöz E, Doğan BN, Aksoy A, Gacar G, Akyüz S, Ayhan S (2010). Isolation and in vitro characterization of dental pulp stem cells from natal teeth. HistochemCell Biol.

[17] Graziano A, d'Aquino R, Cusella-De Angelis MG, De Francesco F, Giordano A, Laino G (2008). Scaffold's surface geometry significantly affects human stem cell bone tissue engineering. J Cell Physiol.

[18] López de Argumedo M, Reviriego E, Andrío E, Rico R, Sobradillo N, Hurtado de Saracho I (2006). Revisión externa y validación de instrumentos metodológicos para la Lectura Crítica y la síntesis de la evidencia científica. Madrid: Plan Nacional para el SNS del MSC. Servicio de Evaluación de Tecnologías Sanitarias del País Vasco.

[19] Atkins D, Eccles M, Flottorp S, Guyatt GH, Henry D, Hill S (2004). Systems for grading the quality of evidence and the strength of recommendations I: Critical appraisal of existing approaches The GRADE Working Group. BMC Health Serv Res.

[20] Kilkenny C, Browne WJ, Cuthill IC, Emerson M, Altman DG (2010). Improving bioscience research reporting: the ARRIVE guidelines for reporting animal research. PLoS Biol.

[21] Moher D, Liberati A, Tetzlaff J, Altman DG, The PRISMA Group (2009). Preferred reporting items for systematic reviews and meta-analyses: The PRISMA statement. PLoSMed.

[22] Cao Y, Liu Z, Xie Y, Hu J, Wang H, Fan Z (2015). Adenovirus-mediated transfer of hepatocyte growth factor gene to human dental pulp stem cells under good manufacturing practice improves their potential for periodontal regeneration in swine. Stem Cell Res Ther.

[23] Gonçalves F, De Moraes MS, Ferreira LB, Carreira AC, Kossugue PM, Boaro LC (2016). Combination of bioactive polymeric membranes and stem cells for periodontal regeneration: In vitro and in vivo analyses. PLoS One.

[24] Hu J, Cao Y, Xie Y, Wang H, Fan Z, Wang J (2016). Periodontal regeneration in swine after cell injection and cell sheet transplantation of human dental pulp stem cells following good manufacturing practice. Stem Cell Res Ther.

[25] Khorsand A, Eslaminejad MB, Arabsolghar M, Paknejad M, Ghaedi B, Rokn AR (2013). Autologous dental pulp stem cells in regeneration of defect created in canine periodontal tissue. J Oral Implantol.

[26] Park JY, Jeon SH, Choung PH (2011). Efficacy of periodontal stem cell transplantation in the treatment of advanced periodontitis. Cell Transplant.

[27] Liu HC, E LL, Wang DS, Su F, Wu X, Shi ZP (2011). Reconstruction of alveolar bone defects using bone morphogenetic protein 2 mediated rabbit dental pulp stem cells seeded on nano- hydroxyapatite/collagen/poly(L-lactide). Tissue Eng Part A.

[28] Yan XZ, Yang F, Jansen JA, de Vries RB, van den Beucken JJ (2015). Cell-based approaches in periodontal regeneration: A systematic review and meta-analysis of periodontal defect models in animal experimental work. Tissue Eng Part B Rev.

